# Evaluating the mosquito host range of Getah virus and the vector competence of selected medically important mosquitoes in Getah virus transmission

**DOI:** 10.1186/s13071-023-05713-4

**Published:** 2023-03-15

**Authors:** Faustus Akankperiwen Azerigyik, Astri Nur Faizah, Daisuke Kobayashi, Michael Amoa-Bosompem, Ryo Matsumura, Izumi Kai, Toshinori Sasaki, Yukiko Higa, Haruhiko Isawa, Shiroh Iwanaga, Tomoko Ishino

**Affiliations:** 1 Department of Parasitology and Tropical Medicine, Tokyo Medical and Dental University, Bunkyo-ku, Tokyo, Japan; 2 Department of Medical Entomology, National Institute of Infectious Diseases, Shinjuku-ku, Tokyo, Japan; 3 Department of Biomedical and Diagnostic Sciences, University of Tennessee, Knoxville, TN USA; 4 Department of Molecular Protozoology, Research Center for Infectious Disease Control, Research Institute for Microbial Diseases, Osaka University, Suita, Osaka Japan

**Keywords:** Arbovirus, Alphavirus, Getah virus, Mosquito-borne, *Culex tritaeniorhynchus*, Vector competence, Susceptibility, Transmission

## Abstract

**Background:**

The Getah virus (GETV) is a mosquito-borne* Alphavirus* (family* Togaviridae*) that is of significant importance in veterinary medicine. It has been associated with major polyarthritis outbreaks in animals, but there are insufficient data on its clinical symptoms in humans. Serological evidence of GETV exposure and the risk of zoonotic transmission makes GETV a potentially medically relevant arbovirus. However, minimal emphasis has been placed on investigating GETV vector transmission, which limits current knowledge of the factors facilitating the spread and outbreaks of GETV.

**Methods:**

To examine the range of the mosquito hosts of GETV, we selected medically important mosquitoes, assessed them in vitro and in vivo and determined their relative competence in virus transmission. The susceptibility and growth kinetics of GETVs in various mosquito-derived cell lines were also determined and quantified using plaque assays. Vector competency assays were also conducted, and quantitative reverse transcription-PCR and plaque assays were used to determine the susceptibility and transmission capacity of each mosquito species evaluated in this study.

**Results:**

GETV infection in all of the investigated mosquito cell lines resulted in detectable cytopathic effects. GETV reproduced the fastest in *Culex tritaeniorhynchus-* and *Aedes albopictus-*derived cell lines, as evidenced by the highest exponential titers we observed. Regarding viral RNA copy numbers, mosquito susceptibility to infection, spread, and transmission varied significantly between species. The highest vector competency indices for infection, dissemination and transmission were obtained for *Cx. tritaeniorhynchus*. This is the first study to investigate the ability of *Ae. albopictus* and *Anopheles stephensi* to transmit GETV, and the results emphasize the role and capacity of other mosquito species to transmit GETV upon exposure to GETV, in addition to the perceived vectors from which GETV has been isolated in nature.

**Conclusions:**

This study highlights the importance of GETV vector competency studies to determine all possible transmission vectors, especially in endemic regions.

**Graphical Abstract:**

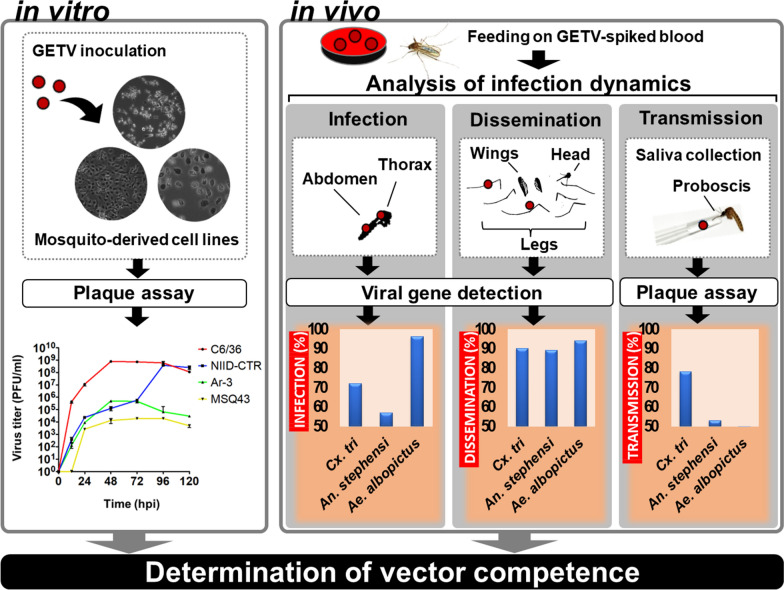

**Supplementary Information:**

The online version contains supplementary material available at 10.1186/s13071-023-05713-4.

## Background

The Getah virus (GETV) is a mosquito-borne virus and a member of the *Alphavirus* genus, one of the two genera that comprise the* Togaviridae* family (the other being the genus *Rubivirus*) [1]. Like other members of this genus, GETV has an icosahedral capsid and a positive-sense, single-stranded RNA with an 11,598-nucleotide-long genome that mimics messenger RNA (mRNA) [1]. There are > 30 species in the *Alphavirus* genus, which can be further divided into seven sera-complexes, including the Barmah Forest, Middleburg, Ndumu, Venezuelan equine encephalitis, Western equine encephalitis and Semliki Forest virus (SFV) serogroups [2–5]. GETV belongs to the SFV serogroup [6] and is geographically restricted to the Old World, with a widespread distribution across Asia [7–12].

GETV is related to other arthritogenic alphaviruses, such as Chikungunya virus (CHIKV), O’nyong’nyong virus and the closely related Ross River virus (RRV) (all pathogenic viruses in humans) [13]. It has been considered to represent a significant risk in veterinary medicine because of the clinical symptoms it elicits, including nasal discharge, fever, rash, edema, lymphadenopathy in horses and abortion in pigs [14, 15]. Despite being closely related to RRV, which is pathogenic to humans [16], the consequences of GETV infection in humans remain unclear [17, 18]. In addition to the increased number of new GETV strains being isolated from insects and animals [17, 19], seroepidemiological findings from a number of studies suggest human exposure, thus highlighting the risks of these viruses to public health, especially in the context of emerging strains with the potential for virulence [17, 20–22].

GETV was first isolated from *Culex gelidus* mosquitoes in Malaysia in 1955 [23] and subsequently found to be endemic to a number of regions where several disease outbreaks were reported in domestic animals, including horses [12, 24], pigs [25, 26], red pandas [27], wild blue foxes [28] and cattle [29]. Outbreaks of GETV have been reported in Japan [24, 25, 30, 31], India [12] and mainland China [26, 28, 29]; all were linked to domestic animal infections without clear information on the vectors involved. GETV strains have also been detected in a variety of other mosquito species, such as *Culex tritaeniorhynchus, Culex vishnui, Culex fuscocephala, Armigeres subalbatus, Anopheles vagus, Anopheles sinensis, Aedes albopictus, Aedes aegypti, Aedes vexans nipponii*, *Mansonia annulifera*, as well as in some unspecified mosquito species [32, 33]. However, it is still unknown how much these insects contribute to GETV transmission; this lack of information is especially relevant for *Anopheles* mosquitoes, which have the ability to adapt to new and urban dwellings. *Anopheles stephensi* mosquitoes are highly endophilic and anthropophilic and have been determined to be susceptible to infection by CHIKV [34]. It is an urban-dwelling species that is wide-spread in Southeast Asia and the Arabian Peninsula [35]. The introduction of these mosquitoes to new areas is partially driven by their resistance to several classes of insecticides and by increases in international travel, factors which make effective control measures more difficult to implement and faciliate spread to newer areas [35, 36]. Although the susceptibility of *An. stephensi* to GETV infection has never been determined, the high abundance of this mosquito species and related species within the genus can represent a potential risk in terms of GETV transmission. *Culex tritaeniorhynchus* and *Ar. subalbatus* are two mosquito species that are widely distributed across Southeast Asia, and multiple isolates of GETV have been identified in these mosquito species [32], especially *Cx. tritaeniorhynchus*, leading to suggestions that this latter mosquito species may be the principal vector of transmission among farm animals in Japan [37]. *Aedes albopictus,* aside from being highly invasive, is also of global concern due to its vectorial competence in transmitting many arboviruses, including dengue virus in Asia [38]. Thus, the abundance and/or high distribution of these mosquito species in areas where GETV is known to circulate, the susceptibility of these mosquito species to GETV and their role in the spread of medically important arboviruses need to be further investigated.

In this context, it is of increasing importance to expand the scope of assessing vector competency to include indigenous mosquito species with the potential to spread GETV in areas endemic to GETV (owing to increased international travel and altering environmental conditions) [35, 36]. Wild boars, horses, and pigs are examples of putative amplifying hosts for GETV; nonetheless, the persistence of GETV outbreaks in specific areas is contingent upon the presence of effective vectors (such as mosquitoes). This further poses a challenge in characterizing the importance of GETV mosquito vectors in nature and forecasting future outbreaks based on the seasonal activity of these mosquitoes.

Prior studies have used mosquito-derived cell culture systems to address the above challenges by analyzing arbovirus infection and replication as a tractable alternative to current in vivo models for assessing vector competencies [39–41]. These methods have transformed arbovirus studies and have provided deeper insights into virus susceptibility and virus-vector host interactions [41, 42]. For example, C6/36 (derived from *Ae. albopictus*), MSQ43 (derived from *An. stephensi*) and AeAe-GH98 (derived from *Ae. aegypti*) cells are now used as cell culture systems for the propagation of many arboviruses [39–41]. Other cell lines, in addition of mosquito cell lines, that have previously been used for the propagation of GETV include CPK (pig kidney), HmLu-1 (hamster lung), Vero (African green monkey kidney) and BHK-21 (hamster kidney) [43, 44]. Until now, the isolation and propagation of GETV have been limited to C6/36 cells [8, 45, 46] with little to no involvement of other mosquito-derived cultured cells. In the present study, we examined the suitability of several mosquito cell lines, namely MSQ43 (derived from *An. stephensi*), NIID-CTR (derived from *Cx. tritaeniorhynchus*) and Ar-3 (derived from *Ar. subalbatus*), in propagating GETV. We also performed an in vivo experiment to evaluate the vector competency of *Cx. tritaeniorhynchus*, *An. stephensi*, and *Ae. albopictus* for transmitting GETV.

## Methods

### Cell lines and GETV strain

Four mosquito-derived cell lines and one mammalian-derived cell line were used in this study. *Culex tritaeniorhynchus*-derived NIID-CTR cells [47] and *Ar. subalbatus*-derived Ar-3 cells [48] were maintained in Varma-Pudney (VP12) medium supplemented with 10% heat-inactivated fetal bovine serum (FBS; Biowest, Nuaillé, France) and penicillin–streptomycin solution (100 U/ml; FUJIFILM Wako Pure Chemical Corp., Osaka, Japan). *Anopheles stephensi*-derived MSQ43 (BEI Resources, Manassas, VA, USA) [49] and *Ae. albopictus*-derived C6/36 cells (European Collection of Authenticated Cell Cultures, Darmstadt, Germany) [50] were cultured in Eagle’s minimum essential medium (MEM) supplemented with 10% heat-inactivated FBS and 2% nonessential amino acids (FUJIFILM Wako Pure Chemical Corp.). One mammalian cell line, Vero cells, derived from the African green monkey kidney (Japanese Collection of Research Bioresources Cell Bank, Osaka, Japan), was maintained in MEM supplemented with 10% heat-inactivated FBS. Mammalian and mosquito cell cultures were adapted to in vitro conditions with 5% CO_2_ and temperatures of 37 °C and 28 °C, respectively*,* except for NIID-CTR cells, which were incubated at 25 °C. The four mosquito-derived cell lines were used for the in vitro growth kinetics study only. Mammalian-derived Vero cells were used for virus stock propagation (prior to in vitro and in vivo experiments) and viral quantification (plaque assay).

The GETV strain used in this study was the 12IH26 strain, isolated from *Cx. tritaeniorhynchus* mosquitoes collected in Isahaya City, Nagasaki Prefecture, Japan, in September 2012 (GenBank accession no. LC152056) [37]. GETV 12IH26, in addition to being recently isolated by our laboratory [37] and being readily available, may be representative of the current and possible circulating strain among farm animals in Japan, as evidenced by recent studies [14].

### Infection of mosquito-derived cell cultures

A monolayer of 1.0 × 10^5^ cells was seeded in a 25-cm^2^ cell culture flask and incubated overnight at 28 °C, 5% CO_2._ The adhered cells were then inoculated with GETV 12IH26 at a multiplicity of infection (MOI) of 0.01 and incubated for 1 h. The cells and cell culture supernatants were harvested at 0, 12, 24, 48, 72, 96 and 120 h post-infection (hpi) and stored at − 80 °C until analysis. The virus titers in cells were determined from freeze-thawed lysates and cell supernatants using the plaque assay method [51]. A plot of virus titers against time was represented by linear regression curves and bar graphs. All samples were run in triplicate with controls (supernatants from mock-infected cell cultures). The effect of GETV infection on cultured cells was determined via microscopy and a Trypan blue-based cell viability test using an automated cell counter (Countess™ II Automated Cell Counter; Thermo Fisher Scientific, Waltham, MA, USA).

### Infection of mosquitoes

Mosquito infections were performed in a BSL-2 insectary at the National Institute of Infectious Diseases, Tokyo, Japan, as previously reported [52, 53]. Briefly, 7- to 14-day-old female mosquitoes were starved overnight and fed defibrinated rabbit blood (Nippon Bio-Supp. Center, Tokyo, Japan) supplemented with 3 mM ATP (Sigma-Aldrich, St. Louis, MO, USA) containing 1.0 × 10^6^ PFU (plaque-forming unit)/ml GETV. Feeding was performed using an artificial membrane feeding system for blood-sucking insects (Hemotek™ 5W1; Hemotek Ltd., Blackburn, UK). Mosquitoes were allowed to feed for 1 h, and only fully engorged mosquitoes were included in subsequent experiments and analyses. Fully engorged females were kept at 27 ± 0.5 °C, and a 10% sugar solution was provided ad libitum; the mosquitoes were maintained for several days before the time points for salivation and dissection.

### Salivation and mosquito dissection

Mosquito salivation and dissection were performed at 5, 10, and 15 days post-infection (dpi) following methods described in previous studies [52, 53]. Briefly, the wings and legs of CO_2_-anesthetized mosquitoes were clipped for immobilization. Salivation was induced by inserting the proboscises of the mosquitoes into 10-µl pipette tips containing 10 µl of FBS. The mosquitoes were allowed to salivate for 1 h prior to dissection. Harvested saliva was collected in tubes containing 100 µl MEM that contained 2% FBS, 2% Amphotericin B (Thermo Fisher Scientific) and 2% penicillin–streptomycin. The mosquitoes were dissected into the thorax/abdomen and head/wings/legs regions, and these regions were stored at − 80 °C until use.

### RNA extraction and virus quantification using quantitative reverse transcription-PCR and plaque assays

RNA was extracted using a Nucleospin RNA extraction kit (Macherey–Nagel, Dueren, Germany) according to the manufacturer’s instructions, with slight modifications. Specifically, prior to extraction, the mosquito body parts were homogenized in a TissueLyser II flexible bead mill (Qiagen, Hilden, Germany) to which RA1 Buffer (Macherey–Nagel) supplemented with 1% beta-mercaptoethanol (FUJIFILM Wako Pure Chemical Corp.) had already been added. The samples were then centrifuged at 12,000 rpm for 3 min; the remaining RNA extraction steps were conducted following the manufacturer’s instructions. The filtered homogenate was subjected to DNase treatment before the RNA was eluted with 20 μl of RNase-free water.

After RNA extraction, quantitative reverse transcription PCR (qRT-PCR) was used to quantify viral copy numbers. Information on the primers and probes used in this study is given in Table 1. Briefly, RNA samples were mixed with Taq-Man Fast Virus 1-Step Master Mix (Thermo Fisher Scientific), GETV primers (forward and reverse, 10 μM each) and GETV probes (10.1 μM), and then quantified by running on a PikoReal RT-PCR System (version 2.2.; Thermo Fisher Scientific). The qRT-PCR cycling conditions were: 1 cycle at 50 °C for 5 min and 95 °C for 20 s, followed by 35 cycles at 95 °C for 3 s and 60 °C for 30 s. Cq values > 30 were regarded as a negative result, as shown in Additional file 1: Figure S1.

**Table 1:** Information on the primer and probe sequences used for quantitative reverse transcription-PCR analysis

Virus	Primer name	Primer sequence (5′–3′)	Nucleotide position
GETV (strain 12IH26)	GETV12IH26 FWD-S	GGAAGCGGTCGTAAATGCTGC	4121–4141
	GETV12IH26 REV-S	GGAACTCCCGTGCTCCAAGG	4692–4671
	GETV12IH26 FWD (Probe)	GGAAGCGGTCGTAAATGCTGC	4121–4141

*GETV *Getah virus

Plaque assays were used to quantify the initial viral stock and mosquito saliva samples. Vero cells were used for plaque assay quantification, as described previously [51]. Briefly, Vero cells were seeded into 24-well plates overnight at a density of 2 × 10^5^ cells/well. A 100-μl aliquot of 1:100 diluted supernatant samples prepared in MEM was added to each well. After incubation for 1 h at 37 °C, the inoculum, MEM supplemented with 1% methylcellulose and 2% FBS were added to each well. The cell setup was further incubated at 37 °C for 3 days, following which the cells were fixed with 4% paraformaldehyde and stained with methylene blue for plaque visualization and PFU estimation.

### Vector competency evaluation points and statistical analysis

The infection rate (IR), dissemination rate (DR) and transmission rate (TR) of all three sample groups (5, 10 and 15 dpi) were computed. The IR was considered to be the proportion of virus-positive mosquito bodies (head-thorax) to fed females ([number of GETV-positive females/total number of fully-fed females]) × 100; the DR was considered to be the proportion of virus-positive mosquito carcasses (head-wings-legs) to virus-positive bodies ([GETV-positive in the carcasses, i.e. head-wings-legs/total number GETV-positive females] × 100). The TR was considered to be the proportion of virus-positive saliva to virus-positive carcasses ([GETV-positive saliva/ GETV-positive carcasses]).

GraphPad Prism software (version 7; GraphPad Software, San Diego, CA, USA) was used for all statistical analyses. Statistical significance was computed between tabulated data points using the Kruskal–Wallis test corrected with Bonferroni’s method and Fisher’s exact test. A *P*-value < 0.05 was considered to be statistically significant. Results are presented as the mean ± standard deviation (SD) [54].

## Results

### GETV growth rate in vitro is species-dependent

**Fig. 1:**
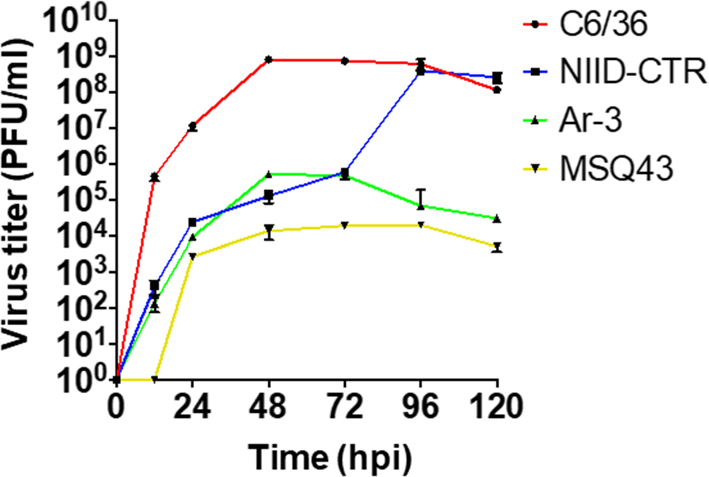
In vitro growth kinetics of Getah virus (GETV) in four mosquito-derived cell lines: NIID-CTR (*Culex tritaeniorhynchus*), C6/36 (*Aedes albopictus*), MSQ43 (*Anopheles stephensi*) and Ar-3 (*Armigeres subalbatus*). Error bars reflect standard deviations (SD) and results are presented as the mean values (SD) from three parallel tests. *PFU* Plaque-forming units

**Fig. 2:**
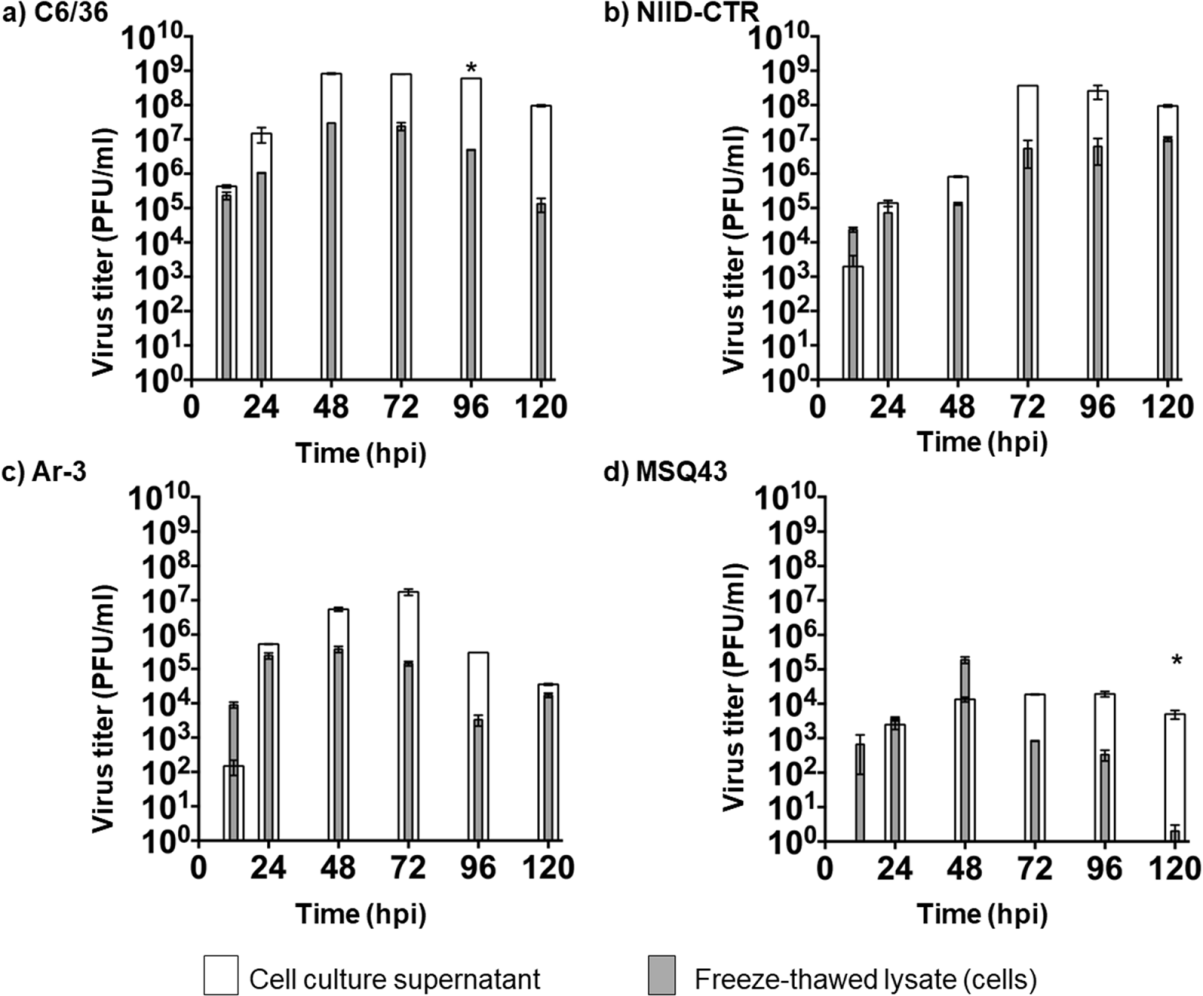
Comparison between the growth titers of GETV in cell culture suspensions and freeze-thawed cell lysates. GETV 12IH26 was used to infect cell monolayers at a multiplicity of infection (MOI) of 0.01, and the cells and supernatants were harvested at 24-h intervals post-infection. Viral titers were measured using Vero cell cultures through the conventional plaque assay and expressed as PFU/well for the cell suspensions and freeze-thawed lysate fractions. **a**, **b**, **c**, and **d** represent GETV growth in C6/36, NIID-CTR, Ar-3, and MSQ43 cell lines, respectively. The error bars show the arithmetic mean and SD of three biological replicates. Statistical significance (*P* < 0.05) was determined using the Kruskal–Wallis test with Bonferroni correction as indicated by asterisks (*). Errors bars represent the median with 95% confidence interval (CI). hpi, Hours post-infection

GETV-infected NIID-CTR (*Cx. tritaeniorhynchus*), C6/36 (*Ae. albopictus*), MSQ43 (*An. stephensi*) and Ar-3 (*Ar. subalbatus*) cells and their respective culture supernatants were collected at 0, 12, 24, 48, 72, 96 and 120 hpi. All four cell lines were found to be susceptible to GETV infection (Fig. 1). In general, the virus titers in the cells were inversely proportional to those in the supernatants in all cell lines, with the exception of NIID-CTR cells (Figs. 1, 2). In terms of GETV propagation efficiency, C6/36 cells were the most efficient cell line for propagating GETV, whereas MSQ43 cells were the least efficient (Fig. 1; Table 2). Similarly, GETV reached the stationary phase of growth within 48 hpi in C6/36 and Ar-3 cells, whereas it took 96 hpi to reach the stationary phase in NIID-CTR and MSQ43 cells (Fig. 1; Table 2). The GETV titers in C6/36 and Ar-3 cells at the plateau phase were 7.87 × 10^8^ and 5.03 × 10^5^ PFU/ml, respectively (Table 2). The highest virus titers were recorded in the extracellular fractions of each cell line over the period 0–120 hpi (Fig. 2). GETV titers in the extracellular fractions generally showed no significant difference compared to the freeze-thawed cell lysates between cell lines despite variations in the peaks at different time points, except in C6/36 (at 96 hpi) and MSQ43 cells (at 120 hpi) (*p* = 0.1467, Fisher’s exact test) (Fig. 2). The virus titers of freeze-thawed lysates of C6/36, NIID-CTR and Ar-3 cells peaked above the initial inoculum titer (10^3^ PFU/ml, 0 hpi) after 12 hpi (10^5^ PFU/ml, Fig. 2a–c). However, MSQ43 cells showed no significant increase in virus titer from the initial inoculum of 10^3^ PFU/ml after 12 hpi (Fig. 2d).

**Table 2:** Sources of mosquito cell lines and estimated peak titers of Getah virus replication in each cell culture

Cell lines	Peak titer (PFU/ml)^a^	Time (hpi)	Source of cell line	References
C6/36	7.87 ± 2.4 × 10^8^	48	Larvae of *Aedes albopictus*	[47]
NIID-CTR	3.80 ± 3.8 × 10^8^	96	Embryos (fertilized eggs) of *Culex tritaeniorhynchus*	[48]
Ar-3	5.03 ± 5.1 × 10^5^	48	Neonate larvae of *Armigeres subalbatus*	[49]
MSQ43	1.93 ± 2.7 × 10^4^	96	First stage larvae of *Anopheles stephensi*	[50]

*hpi* Hours post-infection,* PFU* plaque-forming units

Values for peak titer are given as the mean ± standard deviation (SD)

### GETV proliferation induces the cytopathic effect and decreases cell proliferation

**Fig. 3:**
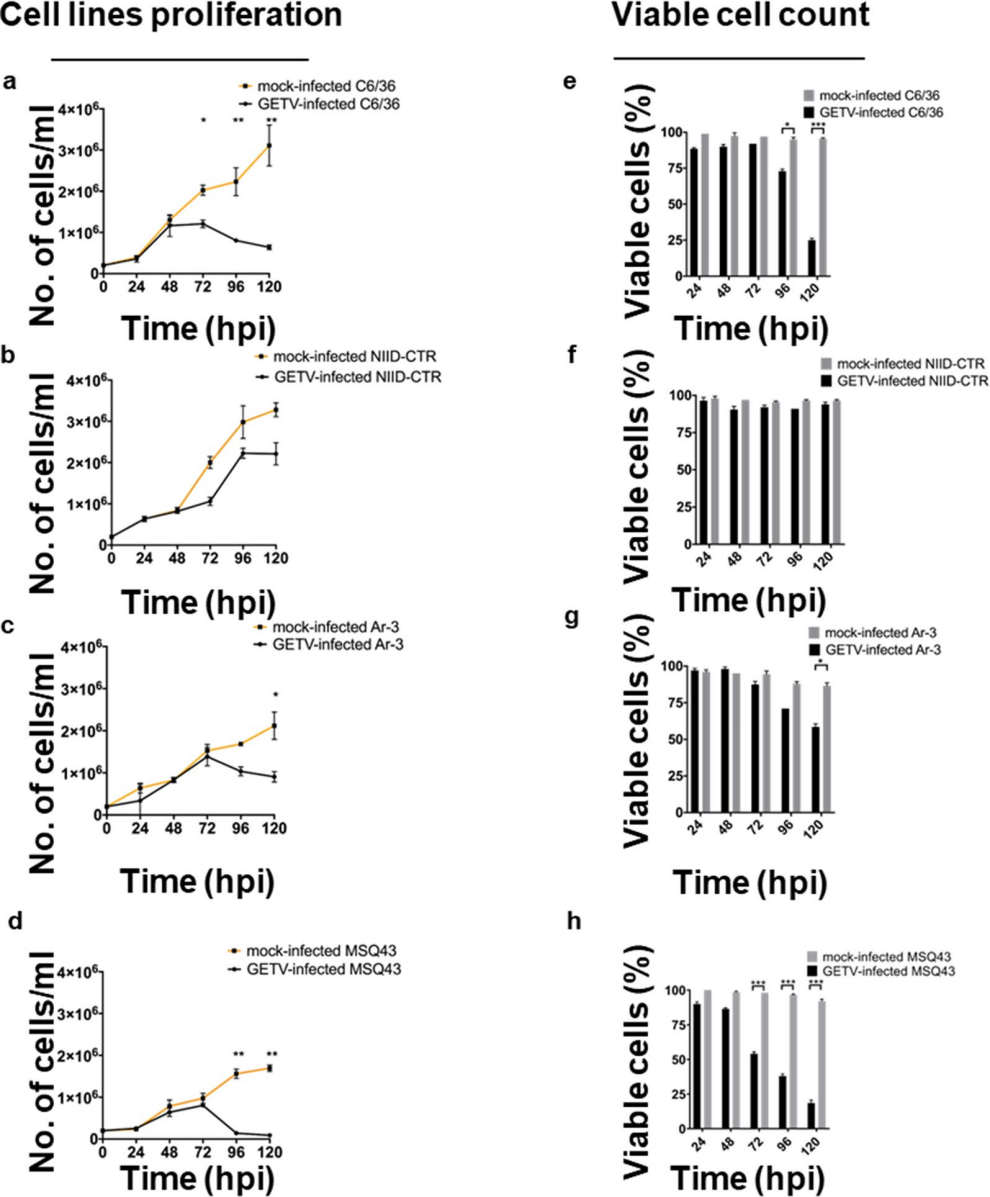
Estimated cell count of mosquito-derived cell lines after GETV infection. **a**–**d** Estimation of cell numbers, **e**–**h** estimation of the percentage viable cell population post-infection with GETV. A MOI of 0.01 was used. Error bars represent the mean with 95% CI. Asterisks indicate statistical significance at *P < 0.05 (significant), **P < 0.01 (very significant) and ***P < 0.001 (extremely significant), using the Kruskal–Wallis test with Bonferroni correction. *No.* Number

The morphological alterations that occurred in the cells were examined, and pictures were taken of the GETV-induced cytopathic effect (CPE). Mock-infected cells were used as the control, and the control cells were compared with GETV-infected cells (Additional file 2: Figure S2). An apparent CPE was observed in GETV-infected C6/36, NIID-CTR, Ar-3 and MSQ43 cells after 48, 72, 72 and 72 hpi, respectively (Additional file 2: Figure S2g, r, bb, ll), with the appearance of some rounded, aggregated and detached cells. An obvious CPE was the decrease in the number of cells, especially in C6/36 and MSQ43 cells at 72 hpi (Additional file 2: Figure S2,h, ll) together with an increase in cell distance. The most severe GETV-induced CPE was observed in MSQ43 cells (120 hpi); in contrast, the least severe CPE was observed in NIID-CTR cells. Characteristic compact cell aggregations were missing in the GETV-infected Ar-3 cells [48]. The mock-infected cells from each cell line demonstrated significant overgrowth, with sloughing and stacking of the cell monolayers, when observed up to 120 hpi (Additional file 2: Figure. S2). This observation was confirmed by estimating the cell population using a Trypan blue exclusion assay (Fig. 3a–d). This assays showed that cell death in GETV-infected cells corresponded to a loss of monolayer integrity over time, with the percentage of viable cells dropping from a brief period of proliferation (Fig. 3e–h), consistent with the cytolysis observed via microscopic analysis at each time point (Additional file 2: Figure S2). A significant decrease in MSQ43 cell proliferation in cell culture was observed following GETV infection (*P* = 0.05) compared to C6/36, NIID-CTR, and Ar-3 cells. In addition, NIID-CTR cells were more resistant to GETV-induced CPE (Fig. 3e–h) despite the relatively high permissiveness of these cells to GETV propagation (Fig. 1).

### GETV specificity in mosquito vectors

**Fig. 4:**
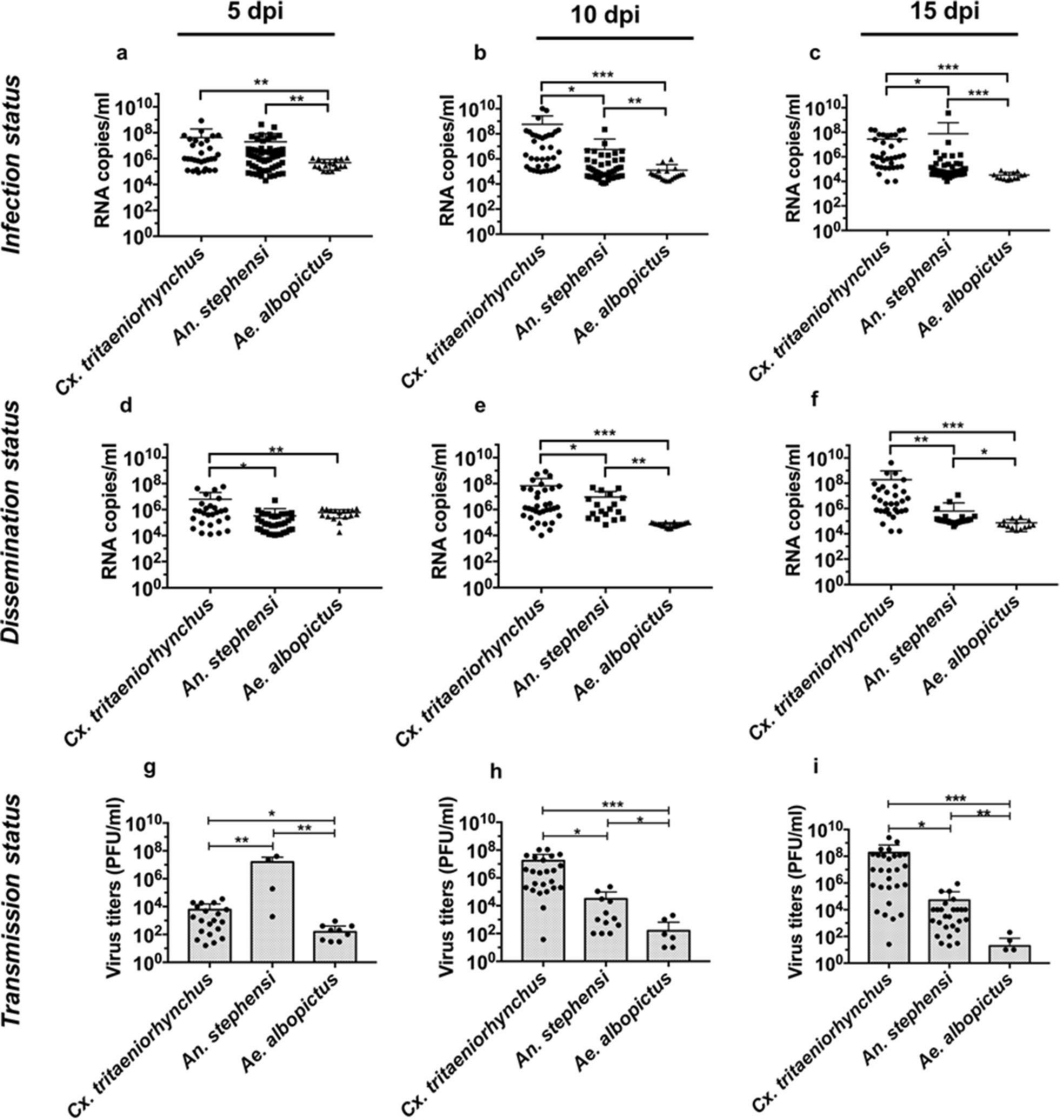
Vector competence of mosquito colonies post-infection. **a**–**c** GETV RNA copy numbers within the thorax and abdomens of GETV-infected mosquitoes, **d**–**f** dissemination of the GETV genome in the heads, wings and legs of GETV-infected mosquitoes, **g**–**i** estimation of live virus titers collected in the saliva of GETV-infected mosquitoes. **a**, **d**, **g** represent 5 dpi, **b**, **e**, **h** represent 10 dpi and **c**, **f**, **i** represent 15 dpi. Each dot in the plots represents an individual specimen. Statically significant variations between GETV viral titers at every time point were determined using Fisher’s exact test; two-tailed *P* = 0.025 was not considered to indicate significance. Asterisks indicate statistical significance at *P < 0.05 (significant), **P < 0.01 (very significant) and ***P < 0.001 (extremely significant). *dpi* Days post-infection

A total of 141 *Cx. tritaeniorhynchus*, 146 *An. stephensi* and 51 *Ae. albopictus* mosquitoes were evaluated. The feeding rates in these mosquito colonies were 78% (141/180), 92% (156/170), and 43% (51/120) for *Cx. tritaeniorhynchus, An. stephensi* and *Ae. albopictus*, respectively. All three tested species were susceptible to GETV infections in their midgut, with the infection established within 5 days of virus acquisition (Table 3; Fig. 4a–c). *Aedes albopictus* was the most susceptible of the four mosquito species to midgut infection, with a combined IR of 96%, while *An. stephensi* was the least susceptible, with a combined IR of 57% (Table 3). Further analysis of viral RNA copy numbers within the midgut of each species after virus exposure showed a significant decrease in viral RNA content (*P* = 0.01, analysis of variance [ANOVA]). In addition, the number of viral RNA copies detected in the midgut of individual mosquitoes per sample decreased with time (Fig. 4a–c). The viral titers in the midgut of *Cx. tritaeniorhynchus* decreased further, while significantly higher (*P* < 0.05) GETV RNA copy numbers were recorded in this species compared to *An. stephensi* and *Ae. albopictus* (Fig. 4a–c). Based on RNA replication data, *Cx. tritaeniorhynchus* was the most susceptible to GETV infection with increasing time post-exposure (Fig. 4a).

**Table 3:** Summary of results of exposure of *Culex tritaeniorhynchus*, *Anopheles stephensi* and *Aedes albopictus* colonies to Getah virus

Source	dpi	Thorax-abdomen	Head-wings-legs	Saliva	Combined		
					Thorax-abdomen	Head-wings-legs	Saliva
		IR	DR	TR	IR	DR	TR
*Cx. tritaeniorhynchus*	5	31/45 (69%)	27/31 (87%)	20/27 (74%)	102/141 (72%)	92/102 (90%)	72/92 (78%)*
	10	36/49 (73%)	34/36 (94%)	24/34 (71%)			
	15	35/47 (74%)	31/35 (89%)	28/31 (90%)			
*An. stephensi*	5	43/60 (72%)*	39/43 (91%)	4/39 (10%)	89/156 (57%)	79/89 (89%)	42/79 (53%)
	10	15/47 (32%)	13/15 (87%)	12/13 (92%)*			
	15	31/49 (74%)*	27/31 (87%)	26/27 (96%)*			
*Ae. albopictus*	5	16/17 (94%)	15/16 (94%)	9/15 (60%)*	49/51 (96%)*	46/49 (94%)	19/46 (41%)
	10	20/20 (100%)	20/20 (100%)	6/20 (30%)			
	15	13/14 (93%)	11/13 (85%)	4/11 (36%)			

The rates of infection, dissemination, and transmission for each of the three mosquito colonies exposed to GETV are contrasted in this table. The data demonstrate the three mosquitoes' suitability as prospective GETV vectors, with *Cx. tritaeniorhynchus* in particular displaying increasing vulnerability to GETV transmission over time (between 5 and 15 dpi). The ratio of positive females to the total number of females examined is shown by the numbers in parenthesis.

*For a given parameter, colonies marked with an asterisk differ significantly at *P* < 0.005 according to the Chi-squared test with Bonferroni correction

*dpi* Days post-infection, *DR* dissemination rate, *IR* infection rate, *TR* transmission rate

### RNA copy numbers differed significantly among mosquito species during dissemination

The dynamics of GETV dissemination were determined in each mosquito species by measuring the amount of viral RNA in the heads, wings and legs of each individual mosquito evaluated (Fig. 4d–f). Virus dissemination within the mosquito colonies post-infection was consistent with their susceptibility to infection. For example, *Cx. tritaeniorhynchus* had significantly higher RNA copy numbers at all time points (*P* = 0.01), which was consistent with the high RNA dissemination recorded with increasing time (Fig. 4d). Generally, there was a decrease in the number of GETV-disseminating individuals and lower GETV RNA titers in *An. stephensi* and *Ae. albopictus* (Fig. 4d–f) relative to previous reports where a higher number of GETV-infected individuals and higher GETV RNA titers post-GETV-laced blood meal (Fig. 4a–c) were observed, respectively. Although a combined DR > 80% was recorded for each colony, the highest sensitivity to virus dispersion per number of individuals was recorded among the *Ae. albopictus* colonies (combined value: 94%, *P* = 0.351; Table 3), followed by *Cx. tritaeniorhynchus* and *An. stephensi* at 90% and 89%, respectively.

### Significant differences in the transmission rate between mosquito species

**Fig. 5:**
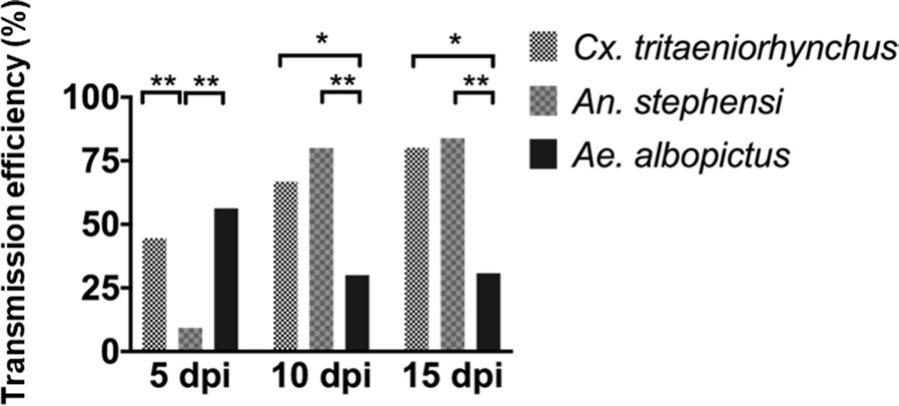
Comparative transmission efficiency (TE) between *Cx. tritaeniorhynchus, An. stephensi* and *Ae. albopictus* colonies. Each bar represents the percentage (%) TE of each mosquito species at different extrinsic incubation periods (5, 10 and 15 dpi). The TE was determined as the proportion of GETV-infected mosquitoes exposed to the infectious blood meal that developed detectable virus titers in their saliva. Statically differences between GETV viral titers at each extrinsic incubation period were determined using Fisher’s exact test;; two-tailed *P* = 0.025 was not considered to indicate significance. Asterisks indicate statistical significance at *P < 0.05 (significant) and **P < 0.01 (very significant)

To ascertain their capacity for GETV transmission, the saliva of mosquitoes that showed signs of virus propagation was investigated for the presence of live viruses. The TR was calculated based on the proportion of mosquitoes with saliva that tested positive for virus among those with positive RNA dissemination. The TRs ranged from 30% to 96% over extrinsic incubation periods of 5, 10, and 15 days. In terms of numbers, *Cx. tritaeniorhynchus* had the highest population to achieve positive TR (74%, 5 dpi; Table 3). The highest titer in GETV-positive individuals (maximum titer: 10^7^ PFU/ml; Fig. 4g) was recorded in *An. stephensi* despite it having the fewest GETV-positive individuals. *Anopheles stephensi* also showed the longest extrinsic incubation period to produce detectable viruses in the saliva, at 10 dpi (96%). In terms of combined TR, *Cx. tritaeniorhynchus* was the colony with the most significant successive transmission (78%, *P* = 0.001), followed by *An. stephensi* and *Ae. albopictus* at 53% and 41%, respectively (Table 3). Live viruses were detected in the saliva of all colonies, indicating the ability of all colonies to produce detectable viruses effectively (Fig. 4g–i). We also observed a decrease in the TR of *Ae. albopictus* from 60% at 5 dpi to 36% at 15 dpi (Table 3). In contrast with* Cx. tritaeniorhynchus* and* An. stephensi*, there was a significantly continuous increase in TR with extension of the extrinsic incubation period (10 and 15 dpi) throughout the testing period (Fig. 4g) compared with TRs in *Ae. albopictus*. Generally, the lowest virus titers in terms of IR, DR and TR were recorded in the *Ae. albopictus* colony (Fig. 4). A comparison of the transmission efficiency (TE), which refers to the proportion of GETV-infected mosquitoes exposed to the infectious blood meal that developed detectable virus levels in their saliva, showed that the *Cx. tritaeniorhynchus* colonies were more efficient (gently increasing slope) in terms of transmission (from 5 to 15 dpi) than the other colonies (Fig. 5).

## Discussion

Beyond RNA detection, only a limited number of studies have investigated the vector range of mosquitoes involved in GETV transmission [32]. Some studies seeking to address this gap were restricted to investigations using *Ae. albopictus-*derived cells, C6/36 and *Cx. tritaeniorhynchus* mosquitoes for in vivo analysis due to the importance of these mosquitoes in natural GETV transmission. In the present study, we have shown that the vector compatibility of GETV can be assessed using a range of mosquito-derived cells and demonstrated the competency of these mosquito species for GETV transmission in vivo.

We examined GETV propagation in mosquito cells by comparing the viral titers and CPE in each cell line and discovered that GETV replication was species-specific in vitro. When the cells were exposed to GETV, we found that the cell line NIID-CTR, derived from *Cx. tritaeniorhynchus*, displayed the most improved susceptibility to GETV replication with minimal pathogenicity, as indicated by the significantly less severe CPE we observed in this cell population. Some arboviruses can interfere with normal cell proliferation by manipulating the subcellular structures of tissues or cells. These viral-induced side effects are frequently characterized by an apparent CPE linked to cell death, such as via apoptosis [55, 56]. Although cytopathology has been observed in some arbovirus-mosquito infections, the apparent but milder CPE observed in NIID-CTR cells suggests that these cells may have evolved to avoid this effect, similar to other host virus-adapted cells [57, 58]. However, it is unclear from the outset whether apoptosis always represents an antiviral response during arbovirus infections in mosquitoes, as we observed that the proliferation of MSQ43 cells was hampered by the replication of GETV. The exposure of C6/36 cells to GETV also resulted in a severe CPE, indicative of the disruptive effect of GETV in these cells. It is important to note that C6/36 cells have defective RNA interference (RNAi) mechanism, implicating the severe CPE observed in these cells when exposed to many other viruses [59–61]. As viral titers increased over time, we also observed that the Ar-3 cell line was susceptible to GETV replication in vitro, especially when the virus titer increased from approximately 10^3^ PFU/ml (initial inoculum) to 10^6^ PFU/ml. Despite the evident CPE in Ar-3 cells, the severity of infection was relatively lower than that in C6/36 cells. The Ar-3 cell line, which was derived from *Ar. subalbatus*, has become an invaluable tool for the titration of some flaviviruses, including the Japanese encephalitis virus, but has a relative insensitivity to another flavivirus, the dengue virus [48]. The detection of GETV in field-sampled *Ar. subalbatus* mosquitoes [9, 28, 32] and the ability of Ar-3 cell lines to support GETV replication, as shown in the current study, provides us with information on its potentially important role in the study of GETV replication and application in GETV isolation in future surveillance studies.

We also explored the potential tractability of our in vitro findings in vivo and evaluated the vector competency of the three mosquito species. Among the colonies tested, the lowest feeding rate was observed in *Ae. albopictus* colonies, which had considerable difficulty feeding under laboratory conditions, as has been reported in previous studies using *Ae. vexans* [62]. In the current study, we compared the IRs of *Cx. tritaeniorhynchus* (72%) and *Ae. albopictus* (96%). The IR showed the ability of the virus to escape the midgut infection barrier and to infect the midgut of the mosquito after exposure to the infected blood meal. To our knowledge, this is the first study that has evaluated the transmission capacity of GETV in laboratory-raised *Ae. albopictus* colonies. Our findings are consistent with previous assessments [62] that showed a higher IR in *Ae. vexans nipponii* (100%) than in *Cx. tritaeniorhynchus* (64%), despite the lower feeding rates among *Aedes* colonies in the previous study [62] and the current study. In *An. stephensi*, a combined IR of 57% was observed. This result is not comparable to that reported in any previously published studies since this is the first reported demonstration of the competency of an *Anopheles* species, *An. stephensi,* to GETV. The IR of *An. stephensi* indicated adequate compatibility with GETV infection in vivo, which corresponds to the ability of *An. stephensi*-derived MSQ43 cells to support GETV replication, albeit with lower titers under in vitro conditions.

TR represented the ability of the mosquito to harbor infectious virus for transmission, i.e. infection of the salivary gland and escape barriers. In terms of TR, *Cx. tritaeniorhynchus* and *An. stephensi* colonies showed a high level of transmissibility, that is, a combined TR of 78% for the former, compared to *Ae. albopictus* colony. A previous study reported a TR between 0 and 59% in *Cx. tritaeniorhynchus*, where GETV titers were determined using plaque assays of viruses derived from mosquito saliva secreted into serum-agar or via mouse feeding [62]. However, it is important to note that these disparate TR outcomes may have been influenced by variations in the inoculating titers used in the blood meal of the mosquitoes [62]. In the current study, the combined TRs of 41 and 53% for *Ae. albopictus* and *An. stephensi*, respectively, were significantly lower (*P* < 0.05) than that of *Cx. tritaeniorhynchus*, which had a combined TR of 78%. A previous study showed that chickens might potentially contract GETV from infected *Cx. tritaeniorhynchus* mosquitoes but not from *Culex pipiens pallens* [63]. These results, along with earlier accounts of GETV isolation from *Culex* mosquitoes [32, 39], led us to speculate that *Cx. tritaeniorhynchus* mosquitoes are highly susceptible to GETV infection and are able to transmit the virus. Notably, the variable TRs of GETV across *Aedes* and *Culex* species have been previously reported in earlier accounts [32, 62], demonstrating differences in TR even among closely related species. 

Among the vectors thought to be involved in the transmission of RRV, a human pathogen and an antigen that closely resembles GETV, *Culex annulirostris, Aedes vigilax, Aedes notoscriptus* and *Aedes camptorhynchus* mosquitoes have been implicated in RRV transmission in nature [64]. Differences in mosquito species that function as vectors of RRV and GETV, despite the serological closeness of these two viruses, may further explain the variations in vector competence in this study. *Culex tritaeniorhynchus* is often perceived to be a major vector of GETV transmission in nature, especially in GETV-endemic regions because: (i) GETV isolates have been detected and isolated in this mosquito species; and (ii) this mosquito species shows increased feeding behavior among large domestic animals [65, 66].

 Interestingly, the TR and TE of the GETV in *An. stephensi* (no reports of GETV competence) was much higher than the TR and TE of GETV in *Cx. tritaeniorhynchus* and *Ae. albopictus* at 10 dpi and 15 dpi, respectively. The extrinsic incubation time also differed among these three species, highlighting the importance of taking into consideration the time needed for each mosquito to become infectious following exposure to GETV. Furthermore, the salivary glands of *Aedes* mosquitoes have been suggested to be potentially affected when exposed to SFV infection, which triggers an effective antiviral response that results in an observable CPE [67]. This finding is significant and should be highlighted as it was previously reported that apoptosis was observed in the salivary glands of *Ae. albopictus* when infected with the Sindbis virus [68, 69], which may affect feeding behavior or reduce virus production in the saliva. In the current study, this phenomenon may explain the low detection rate of infectious viruses in the saliva of *Ae. albopictus* colonies (TR = 41%), as the immunity within the salivary glands of these mosquito colonies may be refractory to GETV replication or detection compared to colonies of *Cx. tritaeniorhynchus* and *An. stephensi.*. The high GETV IR and DR and the ability of the virus to cross the midgut barrier and replicate in the body parts of the mosquito [70], in both *Ae. albopictus* and *An. stephensi*, support the conclusion that GETV can establish infections in the midguts of these species. The low TR of GETV in *Ae. albopictus* may suggest that the salivary gland barrier is refractory to GETV secretion into the saliva, accounting for the lower TR observed in these species. However, *Ae. albopictus* was still able to effectively transmit GETV, emphasizing the significance of the midgut infection barrier as an indicator of vector competence. Additional observational studies focusing on the exposure time, anatomical barriers of the mosquitoes, temperature conditions and immunity within mosquito species are necessary to further characterize and ascertain GETV vector competence. It is noteworthy that although *An. stephensi* mosquitoes have long been regarded as malaria vectors native to the Middle East and South Asia, there have been no reports of GETV detection or transmission perpetuated by *An. stephensi. *However, since *Anopheles* species are known to be robust in their ability to adapt to various needs, coupled with the previous isolation of GETV from *An. sinensis* mosquitoes [9], the spread of indigenous species to new areas, such as the recent discovery of *Anopheles belenrae* in Japan [71], and the potential for emergence of more virulent GETV strains, increases the risk of GETV epidemics.

## Conclusions

Our data suggest that, compared to the three other mosquito species examined, *Cx. tritaeniorhynchus* showed the greatest capacity for the spread and transmission of GETV. The results of this study also confirmed GETV susceptibility in the other mosquito species studied, including *Ae. albopictus* and *An. stephensi*. To the best of our knowledge, this is the first study of GETV infection in *An. stephensi*, and the results highlight the possible role of this species in the GETV transmission cycle. Although GETV growth kinetics in each cell line did not always indicate transmission, the mosquito-derived cell system used in this study offered valuable insights into the susceptibility of mosquito cells and the vector range of GETV. Our aim in the present study was to replicate all in vitro experiments using colonies of the selected mosquitoes; however, current unavailability of a laboratory colony of *Ar. subalbatus* was a limitation in this study. Importantly, this study provides relevant evidence on the different vector species for GETV transmission, as well as recommendations regarding investigations into the sero-related virus RRV.

## Supplementary Information

**Additional file 1: Figure S1. a** Plot showing the TaqMan™ Fast Virus 1-Step Master Mix qRT-PCR output for 1:10 serial dilutions of the reference GETV RNA. GETV one-step proliferation curves were calculated between 1.0 × 10^10^ and 1.0 × 10^1^ copies/µl.** b** A standard curve for GETV RNA was generated using 1:100 serial dilutions. The RNA dilution titers ranged from 1 × 10^10^ to 1 × 10^1^. The equation derived from the quantitative real-time PCR assay was *y* = – 0.284*x* + 11.876, with* R*^2^ = 0.9997 and efficiency = 98.27. The Cq value was plotted on the* y*-axis, and the viral titers corresponding to the template RNA were plotted on the* x*-axis as log values.

**Additional file 2: Figure S2.** Cell culture characterization of GETV infection and the morphological development of mosquito cell lines via microscopy analysis.** Panels** **a**-**e** represent the proliferation of uninfected *Ae. albopictus*-derived C6/36 and mock-infected C6/36 cells at each time point in hours (hpi).** Panels f**-**j** represent the proliferation of GETV-infected C6/36 cells at each time point after GETV infection.** Panels k**–**o** represent the proliferation of *Cx. tritaeniorhynchus*-derived NIID-CTR and mock-infected NIID-CTR cells at each time point.** Panels m**-**q** represent the proliferation of NIID-CTR cells at each time point post-GETV infection.** Panels r**-**v** represent the proliferation of uninfected *Ar. subalbatus*-derived Ar-3 and mock-infected Ar-3 cells at each time point.** Panels w**-**aa** represent the proliferation of Ar-3 cells at each time point after GETV infection.** Panels bb**-**ff** represent the proliferation of uninfected *An. stephensi*-derived MSQ43 and mock-infected MSQ43 cells at each time point.** Panels gg**-**kk** represent the proliferation of MSQ43 cells at each time point after GETV infection. hpi, Hours post-infection.

## Data Availability

The corresponding author can provide the datasets used and/or analyzed during the current investigation upon reasonable request.

## Abbreviations

CPE: Cytopathic effect
DR: Dissemination rate
GETV: Getah virus
IR: Infection rate
PFU: Plaque-forming unit
qRT-PCR: Quantitative reverse transcription-PCR
RNAi: Ribonucleic acid interference
TE: Transmission efficiency
TR: Transmission rate
