# Supplementary figures and images for: Evaluating the mosquito host range of Getah virus and the vector competence of selected medically important mosquitoes in Getah virus transmission
Source: Parasit Vectors. 2023 Mar 15;16:99. doi: 10.1186/s13071-023-05713-4 (PMC10015795; doi:10.1186/s13071-023-05713-4)

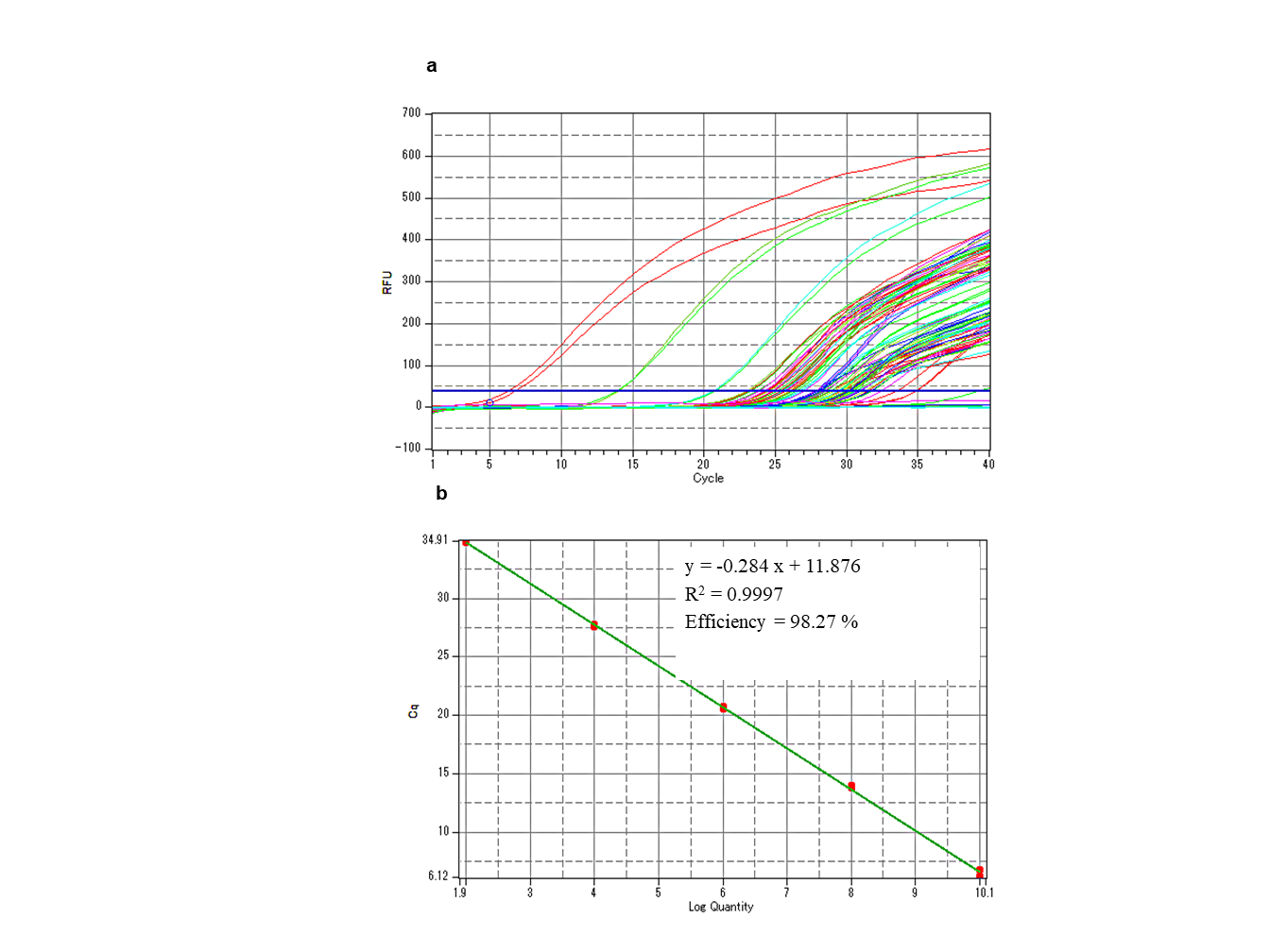

Supplement: Supplementary file 1 — Additional file 1: Figure S1. a Plot showing the TaqMan™ Fast Virus 1-Step Master Mix qRT-PCR output for 1:10 serial dilutions of the reference GETV RNA. GETV one-step proliferation curves were calculated between 1.0 × 10^10^ and 1.0 × 101 copies/µl. b A standard curve for GETV RNA was generated using 1:100 serial dilutions. The RNA dilution titers ranged from 1 × 10^10^ to 1 × 101. The equation derived from the quantitative real-time PCR assay was y = – 0.284x + 11.876, with R2 = 0.9997 and efficiency = 98.27. The Cq value was plotted on the y-axis, and the viral titers corresponding to the template RNA were plotted on the x-axis as log values. [file 13071_2023_5713_MOESM1_ESM.tif]

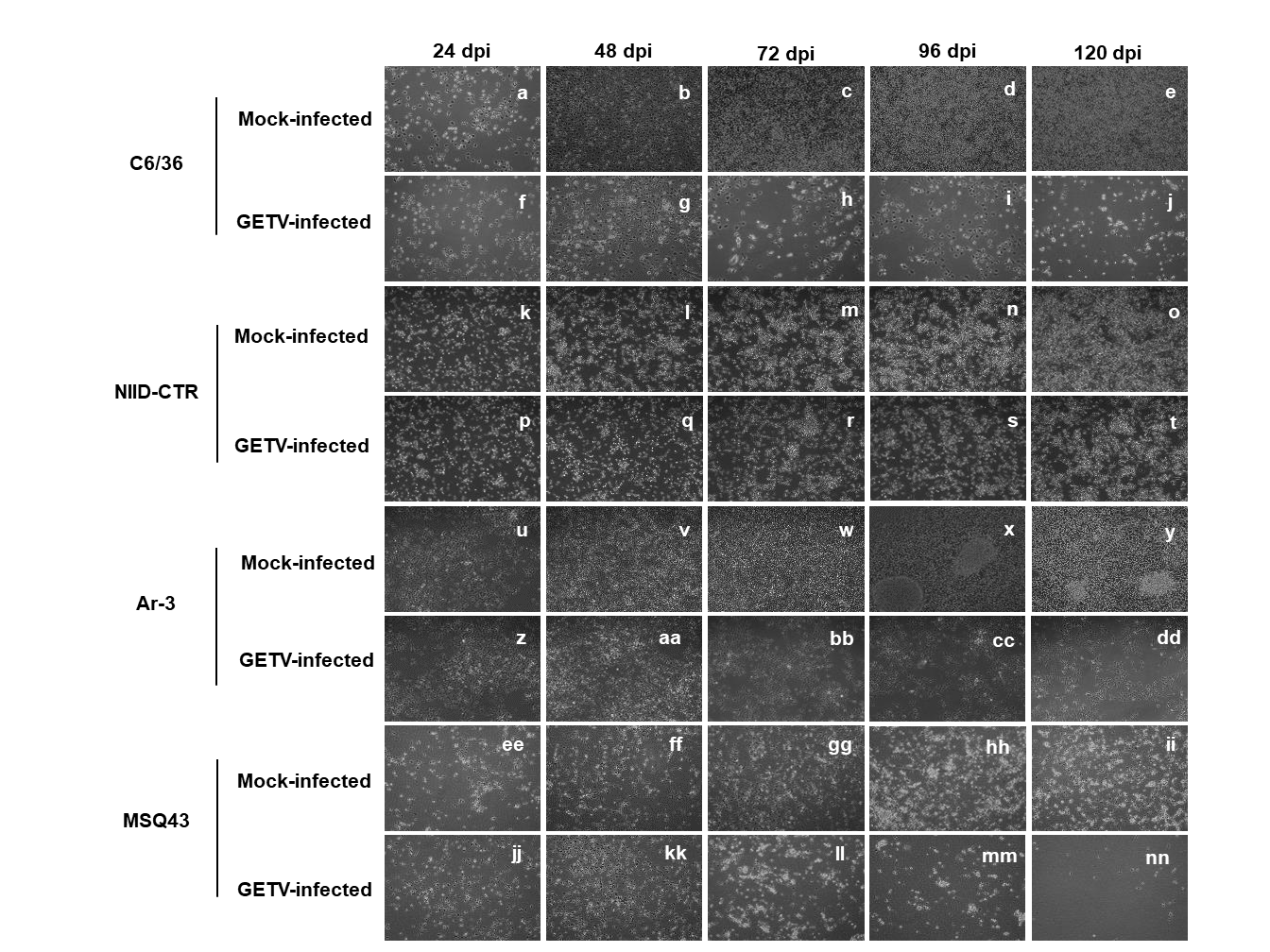

Supplement: Supplementary file 2 — Additional file 2: Figure S2. Cell culture characterization of GETV infection and the morphological development of mosquito cell lines via microscopy analysis. Panels a-e represent the proliferation of uninfected Ae. albopictus-derived C6/36 and mock-infected C6/36 cells at each time point in hours (hpi). Panels f-j represent the proliferation of GETV-infected C6/36 cells at each time point after GETV infection. Panels k–o represent the proliferation of Cx. tritaeniorhynchus-derived NIID-CTR and mock-infected NIID-CTR cells at each time point. Panels m-q represent the proliferation of NIID-CTR cells at each time point post-GETV infection. Panels r-v represent the proliferation of uninfected Ar. subalbatus-derived Ar-3 and mock-infected Ar-3 cells at each time point. Panels w-aa represent the proliferation of Ar-3 cells at each time point after GETV infection. Panels bb-ff represent the proliferation of uninfected An. stephensi-derived MSQ43 and mock-infected MSQ43 cells at each time point. Panels gg-kk represent the proliferation of MSQ43 cells at each time point after GETV infection. hpi, Hours post-infection. [file 13071_2023_5713_MOESM2_ESM.tif]
